# The association between higher education attendance and common mental health problems among young people in England: evidence from two population-based cohorts

**DOI:** 10.1016/S2468-2667(23)00188-3

**Published:** 2023-09-28

**Authors:** Tayla McCloud, Strahil Kamenov, Claire Callender, Glyn Lewis, Gemma Lewis

**Affiliations:** a Division of Psychiatry, Faculty of Brain Sciences, University College London, London, UK; b Department of Psychosocial Studies, Birkbeck University and Institute of Education, University College London, London, UK

## Abstract

**Background:**

It is unclear whether young people who attend higher education are at increased risk of common mental disorders, compared with those who do not attend. We aimed to investigate whether higher education attendance was associated with increased symptoms of common mental disorders (depression and anxiety) in young people before, during and after attendance.

**Methods:**

For this cohort study, we used two cohorts—the Longitudinal Studies of Young People in England (LSYPE1: N=4832, 55·8% [2696 of 4832] students; LSYPE2: n=6128, 50·7% [3104 of 6128] students), beginning in 2004 for LSYPE1 and 2013 for LSYPE2. Both cohorts were designed to be nationally representative, with schools in England as the primary sampling unit. Symptoms of common mental disorders were assessed with the General Health Questionnaire (GHQ-12) before (age 14–17 years for both cohorts), during (age 18–19 years for LSYPE2), and after (age 25 years for LSYPE1) higher education. We assessed differences in GHQ scores using unadjusted and confounder adjusted linear regression.

**Findings:**

At ages 18–19 years (LSYPE2), mean GHQ-12 scores were 12·0 (SD 6·4) among students and 11·6 (SD 6·8) among non-students (adjusted mean difference 0·36, 95% CI 0·05 to 0·68; p=0·024). In LSYPE1, young people who attended higher education at ages 18–20 years had higher symptoms of common mental disorders at ages 16–17 years than those who did not (0·60, 0·30 to 0·90). However, after higher education (age 25 years for LSYPE1), there was no evidence of a difference—mean GHQ-12 scores were 11·4 (SD 5·5) among those who had attended and 11·7 (SD 6·4) among those who had not attended (–0·25, –0·66 to 0·16; p=0·23).

**Interpretation:**

We found evidence that students had more symptoms of common mental disorders than non-students at ages 18–19 years, albeit the effect size was small and there was no evidence of a longer-term difference at the age of 25 years.

**Funding:**

Department for Education.

## Introduction

Depression and generalised anxiety disorder are the two most common mental health conditions. Incidence increases sharply during adolescence (around the age of 12–13 years in girls and 16 years in boys) and continues to rise into the twenties.^1^ There is evidence that rates of these common mental disorders are rising and prevention is a public health priority.^2^

The number of young people attending higher education has increased substantially in the UK^3^ and the proportion of students disclosing mental health conditions has risen six-fold.^4^ However, it is unclear whether young people who attend higher education are at increased risk of common mental disorders compared with those who do not attend higher education. On average, young adults who attend higher education have more highly educated parents with higher incomes than young adults who do not attend higher education. Consistent with evidence on the socioeconomic gradient of mental health,^5^ students could be expected to have better mental health than non-students.^6^ However, students could experience more academic, social, and financial stressors while being in higher education.^7^, ^8^ Potential stressors include academic pressure, burnout, loneliness, decreased social support, and financial strain, which have been found to increase symptoms of depression and anxiety.^8^, ^9^, ^10^, ^11^ The financial and educational benefits of higher education could reduce the risk of mental health problems, but are usually experienced some years after higher education.

Few studies have compared common mental disorders in young people who attended higher education with those who did not. Some studies collected data from students and compared findings with national surveys. However, this comparison is susceptible to bias, and the studies have drawn varying conclusions.^6^, ^12^, ^13^ We need robust comparisons between students and non-students to provide evidence on whether higher education might be a potential cause of mental health problems. Evidence on potential causality is necessary to inform public health and policy interventions.

**Research in context**

**Evidence before this study**

We searched PubMed, the American Psychological Association, PsycInfo, PsycArticles, and CINAHL databases on Jan 30, 2023, for studies that compared symptoms of common mental disorders in young people who had attended higher education with those who had not attended. We used approved Medical Subject Headings terms (((((higher education) OR (university)) OR (students)) AND (depress)) OR (anx)) OR (common mental health), and included studies published in English from database inception to Jan 30, 2023. Evidence on the association between higher education attendance and common mental health problems was inconsistent, and most studies had methodological limitations that might have led to bias. Several studies did not directly compare young people who attended higher education with those who did not attend, and student status was often not measured robustly. Many studies adjusted for a limited set of confounders. It was rare for studies to follow the trajectory of symptoms in the same individuals over time, and most studies were cross-sectional. There had been no investigation of the longer-term mental health outcomes of those who had attended higher education compared with the rest of the population once they had transitioned to adulthood.

**Added value of this study**

We used data from two nationally representative cohorts—the Longitudinal Studies of Young People in England (LSYPE1 and LSYPE2). LSYPE1 and LSYPE2 were conducted 9 years apart, beginning in 2004 for LSYPE1 and 2013 for LSYPE2. We used these data to investigate whether higher education was associated with increased symptoms of common mental disorder in young people before, during, and after attendance. Young people who attended higher education experienced more symptoms of common mental disorders than those who did not attend during higher education at age 18–19 years (in LSYPE2), although the effect size was small. There was no evidence of a difference in common symptoms of mental disorders after higher education at the age of 25 years (in LSYPE1). In LSYPE1, young people who attended higher education at ages 18–20 years had more symptoms of common mental disorders at ages 16–17 years than those who did not attend. This effect size was also small.

**Implications of all the available evidence**

We found evidence that students had more symptoms of common mental disorders than non-students at age 18–19 years, although the effect size was small and there was no evidence of a longer-term difference at the age of 25 years. Our findings are inconsistent with previous studies, which either found no evidence of a difference or evidence of better mental health among students, but this discrepancy might be due to differences in study design, setting, and study year. Our findings suggest that future studies comparing mental health in students with non-students are warranted to investigate the possibility that higher education environments could cause an increased risk of mental health problems among young people.

Some studies directly compared students with the rest of the population in the same study, but have mixed findings and methodological limitations. One survey of young people included data from Australia, Germany, Northern Ireland, and the USA, among others (but not England).^14^ University students had a lower 12-month prevalence of generalised anxiety disorder and depression compared with non-students. One Australian study used three representative surveys^15^ and found no evidence of a difference in symptoms of common mental disorders between students and non-students. A follow-up study found that students in higher education had better mental health than non-students before higher education at the age of 15 years and during their studies at age 18 years.^16^ These studies might not be generalisable to England due to cross-national differences in education systems that are likely to affect mental health.

We are aware of only two studies that directly compared the mental health of students with those who were non-students using representative data from the UK.^17^, ^18^ One study found no evidence that symptoms of depression and anxiety, suicide attempts, or self-harm differed between students in higher education and non-students. The other study found that students in higher education had fewer depressive and anxiety symptoms than non-students. Both studies used data from cross-sectional surveys rather than following the trajectory of symptoms in the same individuals over time. There has also been no investigation of the mental health outcomes in students from higher education compared with non-students once they transitioned to adulthood.

We aimed to investigate whether higher education was associated with increased symptoms of common mental disorders in young people, during and after attendance. We also explored whether young people who attended higher education had more symptoms of common mental disorders during secondary school compared with those who did not.

## Methods

### Study design and participants

For this study, we used data from the Longitudinal Studies of Young People in England—the Longitudinal Study of Young People in England (LSYPE1) and the Longitudinal Study of Young People in England: cohort 2 (LSYPE2). LSYPE1 began in 2004, recruiting participants aged 13–14 years born between 1989 and 1990. Participants were surveyed annually until the age of 19–20 years and again at 25 years in 2015. LSYPE2 (from 2013 to present), with participants born between 1998 and 1999, were surveyed annually until 2019. In both cohorts, parents were interviewed when the participants were aged (waves 1–4). One child was recruited per household.

Both cohorts were designed to be nationally representative. Schools were the primary sampling unit with over-sampling from deprived schools. Pupils within schools were then sampled, with ethnic minority groups over-sampled. The initial LSYPE1 sample contained 15 770 of 21 000 young people (75% of those initially invited). An additional 352 participants were recruited at the age of 16–17 years (wave 4) to increase the number of ethnic minority participants, giving a sample of 16 122. The LSYPE2 sample at wave 1 contained 13 100 young people of 18 290 (72% of those initially invited). Further information is available on the CLS and CLOSER websites.

Ethical approval for LSYPE1 was from the NHS Research Ethics Committee (REC Reference 14/LO/0096) and ethical approval for LSYPE2 was from the Department for Education. Consent was received from all participants, as well as from a parent or guardian.

### Procedures and outcomes

Higher education was defined as studying at a university, higher education college, university college, or private college for a degree or any other undergraduate higher education qualification, including teacher training (BEd, BA, or BSc with QTS), Higher Education Diploma (DipHE), and qualifications at an equivalent level (eg, National Vocational Qualification [NVQ] at level 4 or 5).^19^ To increase statistical power and precision, we used a binary variable indicating whether young people were attending higher education or not. We used the group not attending higher education as the reference category. In LSYPE1, higher education was measured at ages 18–19 years and 19–20 years (waves 6 and 7), and combined into one binary variable indicating whether young people were at higher education at either of these timepoints. In LSYPE2, higher education was measured at age 18–19 years (wave 6) only. We conducted sensitivity analyses with a four-category exposure: (1) studying for a higher education degree; (2) studying in higher education but for a qualification other than degree (eg, teacher training, higher apprenticeship, or diploma); (3) working or training (ie, not in higher education); and (4) not in education, employment, or training. Consistent with the main analyses, we used the group not in higher education who were working or training as the reference category (as this group was larger than those who were not in education, employment, or training).

Symptoms of common mental disorders were measured using the self-administered 12-item General Health Questionnaire (GHQ-12).^20^ Factor analyses of the GHQ-12 show that it measures symptoms of depression, anxiety, and social dysfunction, with the overall score reflecting symptoms of common mental disorders.^20^, ^21^, ^22^ The GHQ-12 is widely used in population-based studies and has high reliability (Cronbach's α 0·82–0·86),^23^ and high sensitivity and specificity for detecting clinical diagnoses of major depression and anxiety.^20^ Respondents indicated how often they experienced each item over the past few weeks. Items were scored on a scale of 0–3, with total scores of 0–36 and higher scores indicating more severe symptoms. We conducted sensitivity analyses with a binary outcome using a bimodal scoring (0, 0, 1, 1), to identify potentially meeting clinical diagnostic criteria for common mental disorders.^24^ Each item was coded as 0 or 1 and a total score of 3 or more indicated common mental disorders.

The timepoints when the GHQ-12 was administered is shown in the appendix (p 11). From here on, we refer to LSYPE2 first because the outcome was measured earlier in the life-course. To investigate the association between higher education and symptoms of common mental disorder during higher education, we used GHQ-12 scores at age 18–19 years (wave 6) in LSYPE2. These scores were collected during spring, after students had started higher education the previous autumn. There was therefore temporality between exposure and outcome. To investigate associations between higher education and symptoms of common mental disorders after higher education we used GHQ-12 scores in LSYPE1 that were collected at age 25 years (wave 8), 5–7 years after young people might have started higher education (at 18–19 years or 19–20 years). To investigate whether symptoms of common mental disorders differed between groups before they attended higher education, we used GHQ-12 scores at ages 14–15 years (wave 2) and 16–17 years (wave 4) in both cohorts and at age 17–18 years (wave 5) in LSYPE2.

For the association between higher education and symptoms of common mental disorders during and after higher education, we selected confounders that were likely to be associated with exposure and outcome but unlikely to be on the causal pathway between them, based on existing evidence and theoretical assumptions. Variables were taken from the same timepoint as the exposure or as close as possible to it, and were as follows: sex; ethnicity; parents’ socioeconomic status (based on the parent with the highest employment category); parents’ highest qualification; family composition; antisocial behaviour in the past 12 months (at age 15–16 years); whether the student experienced bullying in the past 12 months (at age 15–16 years); frequency of alcohol use (at age 16–17 years); if the student ever used cannabis (at age 16–17 years); carer status (at age 16–17 years in LSYPE2, and 16–17 years or 17–18 years in LSYPE1); general quality of health (at age 16–17 years); and disability status (at age 16–17 years). We also adjusted for previous symptoms of common mental disorders to reduce the possibility of reverse causation. In LSYPE2, previous symptoms of common mental disorders were available at age 17–18 years (wave 5) and in LSYPE1 at age 16–17 years (wave 4).

In terms of the association between higher education and common symptoms of mental disorders before higher education, in these analyses, we adjusted for only for sex, ethnicity, parents’ socioeconomic status (based on the parent with the highest employment category), parents’ highest qualification, and family composition. Several of the variables in this analysis were assessed during secondary school, before the exposure. We did not include confounders that were measured after the first outcome assessment to avoid introducing potential collider bias.^25^ Details on how confounders were measured can be found in table 1 and in the appendix (pp 3–6).

**Table 1:** Demographic characteristics of the cohorts stratified by higher education attendance

		**LSYPE2**			**LSYPE1**		
		Attended higher education*	Did not attend higher education*	**Total (N=6128)**	Attended higher education*	Did not attend higher education*	**Total (N=4832)**
Sex†							
	Female	1731 (55·8%)	1509 (49·9%)	3240 (52·9%)	1530 (56·8%)	1084 (50·7%)	2614 (54·1%)
	Male	1373 (44·2%)	1515 (50·1%)	2888 (47·1%)	1166 (43·2%)	1052 (49·3%)	2218 (45·9%)
Ethnicity‡							
	White	2255 (72·7%)	2497 (82·6%)	4752 (77·5%)	1830 (67·9%)	1724 (80·7%)	3554 (73·6%)
	Mixed	135 (4·4%)	125 (4·1%)	260 (4·2%)	105 (3·9%)	102 (4·8%)	207 (4·3%)
	Indian	103 (3·3%)	56 (1·9%)	159 (2·6%)	275 (10·2%)	57 (2·7%)	332 (6·9%)
	Pakistani	136 (4·4%)	85 (2·8%)	221 (3·6%)	151 (5·6%)	95 (4·4%)	246 (5·1%)
	Bangladeshi	112 (3·6%)	48 (1·6%)	160 (2·6%)	123 (4·6%)	65 (3·0%)	188 (3·9%)
	Black African	170 (5·5%)	81 (2·7%)	251 (4·1%)	77 (2·9%)	20 (0·9%)	97 (2·0%)
	Black Caribbean	80 (2·6%)	86 (2·8%)	166 (2·7%)	57 (2·1%)	46 (2·2%)	103 (2·1%)
	Other	113 (3·6%)	46 (1·5%)	159 (2·6%)	78 (2·9%)	27 (1·3%)	105 (2·2%)
Parents' socioeconomic status§¶							
	Managerial and professional occupations	1694 (54·6%)	1239 (41·0%)	2933 (47·9%)	1557 (57·8%)	783 (36·7%)	2340 (48·4%)
	Intermediate occupations	686 (22·1%)	728 (24·1%)	1414 (23·1%)	456 (16·9%)	471 (22·1%)	927 (19·2%)
	Lower supervisory, routine occupations, and not currently working	724 (23·3%)	1057 (35·0%)	1781 (29·1%)	683 (25·3%)	882 (41·3%)	1565 (32·4%)
Parents' highest qualification¶‖							
	Degree or equivalent	670 (21·6%)	335 (11·1%)	1005 (16·4%)	815 (30·2%)	224 (10·5%)	1039 (21·5%)
	Higher education below degree level	440 (14·2%)	278 (9·2%)	718 (11·7%)	536 (19·9%)	319 (14·9%)	855 (17·7%)
	GCE, A Level, or equivalent	421 (13·6%)	345 (11·4%)	766 (12·5%)	445 (16·5%)	419 (19·6%)	864 (17·9%)
	GCSE grades A–C or equivalent	1126 (36·3%)	1429 (47·3%)	2555 (41·7%)	485 (18·0%)	646 (30·2%)	1131 (23·4%)
	Below GCSE or no qualification	447 (14·4%)	637 (21·1%)	1084 (17·7%)	415 (15·4%)	528 (24·7%)	943 (19·5%)
Family composition¶							
	Married or cohabiting	2456 (79·1%)	2169 (71·7%)	4625 (75·5%)	2252 (83·5%)	1530 (71·6%)	3782 (78·3%)
	Lone parent or no parents in the household	648 (20·9%)	855 (28·3%)	1503 (24·5%)	444 (16·5%)	606 (28·4%)	1050 (21·7%)
Antisocial behaviour (in past 12 months)**††		170 (5·5%)	299 (9·9%)	469 (7·7%)	299 (11·1%)	465 (21·8%)	764 (15·8%)
Experienced bullying (in past 12 months)††		839 (27·0%)	970 (32·1%)	1809 (29·5%)	642 (23·8%)	645 (30·2%)	1287 (26·6%)
Frequency of alcohol use‡‡§§							
	Never	1094 (35·2%)	893 (29·3%)	1987 (32·4%)	653 (24·2%)	380 (17·8%)	1033 (21·4%)
	Once every couple of months or less	1189 (38·3%)	1297 (42·9%)	2486 (40·6%)	486 (18·0%)	379 (17·7%)	865 (17·9%)
	1–3 times a month	718 (23·1%)	682 (22·6%)	1400 (22·8%)	893 (33·1%)	636 (29·8%)	1529 (31·6%)
	Once a week or more	103 (3·3%)	152 (5·0%)	255 (4·2%)	664 (24·6%)	741 (34·7%)	1405 (29·1%)
	Cannabis use§§ (ever)	554 (17·9%)	771 (25·5%)	1325 (21·6%)	673 (25·0%)	785 (36·8%)	1458 (30·2%)
General quality of health§§							
	Very good	1363 (43·9%)	1127 (37·3%)	2490 (40·6%)	1488 (55·2%)	1031 (48·3%)	2519 (52·1%)
	Fairly good	1543 (49·7%)	1560 (51·6%)	3103 (50·6%)	1064 (39·5%)	928 (43·4%)	1992 (41·2%)
	Not very good or not good at all	198 (6·4%)	337 (11·1%)	535 (8·7%)	144 (5·3%)	177 (8·3%)	321 (6·6%)
Disability status§§		286 (9·2%)	412 (13·6%)	698 (11·4%)	163 (6·0%)	177 (8·3%)	340 (7·0%)
Carer status¶¶		125 (4·0%)	180 (6·0%)	305 (5·0%)	203 (7·5%)	165 (7·7%)	368 (7·6%)

Data are n (%). Data are unweighted. A Level=Advanced Level. GCE=General Certificate of Education. GCSE=General Certificate of Secondary Education.

* Indicates whether a young person was attending higher education at ages 18–19 years (wave 6) in LSYPE2, or at ages 18–19 years or 19–20 years (wave 6 or 7) in LSYPE1.

† Measured at ages 13–14 years (wave 1) in LSYPE2 and 18–19 years (wave 6) in LSYPE1.

‡ Measured at ages 13–14 (wave 1) in LSYPE2 (missing data supplemented with ages 14–15 years data) and 16–17 years (wave 4) in LSYPE1.

§ Parents' socioeconomic status is based on the socioeconomic status of whichever parent (mother or father) has the highest employment category.

¶ Measured at ages 13–14 (wave 1) in LSYPE2 and ages 16–17 years (wave 4) in LSYPE1.

‖ Indicates the qualification held by whichever parent has the highest qualification. In LSYPE2, the mother or father, and in LSYPE1, the main or second parent.

** In LSYPE2, antisocial behaviour includes taking part in any of the following: damaging anything in a public place on purpose that does not belong to them, shoplifting, graffitiing anywhere, and hitting or attacking someone on purpose with or without using an object or weapon. In LSYPE1, it includes any of the following: vandalising public property, shoplifting, graffitiing on walls, and fighting or public disturbance.

†† Measured at ages 15–16 years (wave 3) in LSYPE2 and LSYPE1.

‡‡ Categories differed slightly from stated at LSYPE2, as follows: never, once a month or less, 2–3 times a month, and two or more times a week.

§§ Measured at ages 16–17 years (wave 4) in LSYPE2 and LSYPE1.

¶¶ In LSYPE2, indicates whether the young person has been a carer at ages 16–17 years (wave 4) only. In LSYPE1, indicates whether the young person has been a carer at ages 16–17 years (wave 4) or ages 17–18 years (wave 5).

### Statistical analysis

For the association between higher education and symptoms of common mental disorders during and after higher education, we conducted analyses separately for LSYPE1 and LSYPE2 using linear regressions. We ran univariable models and then incrementally added confounders to observe their influence on associations between exposure and outcome. In this analysis, participants were included if they had complete data on all variables used in each analysis (appendix pp 7–10) and survey weights were used to re-weight the sub-sample analysed so it represented the target population. In line with the Longitudinal Surveys of Young People in England guidance, we selected weights from the same timepoint as the outcome.^26^ In both analyses, we examined whether associations differed according to ethnicity or parents’ education (whether the participants’ parents had a degree), by assessing interaction terms. To increase power and precision, we collapsed the ethnicity variable to White, Asian, Black, or Mixed or other ethnicity.

For the association between higher education and symptoms of common mental disorders before higher education, we used multilevel linear regressions to model the trajectories of symptoms of common mental disorders among individuals who attended higher education compared with those who did not. First, we ran a univariable model, and next, we added a categorical variable for time. We then tested for a non-linear association with time using a quadratic time variable. If there was evidence of non-linearity, the quadratic time variable was retained. We then tested whether associations between higher education and GHQ-12 scores differed by time using interactions between higher education and linear and quadratic time variables. We then adjusted for confounders. Where there was evidence of a difference by time, associations were presented separately by timepoint. We did not report p values by timepoint because p values from subgroup analyses can be unreliable.^27^ For these analyses participants were included if they had complete data on exposure, confounders, and at least one GHQ-12 score.

We conducted several sensitivity analyses for the main analysis (appendix pp 12–15). We analysed GHQ-12 as a binary outcome, we repeated analyses excluding participants in LSYPE1 who took a gap year after secondary school and before higher education (people who took a gap year were not included in LSYPE2), we repeated the analyses with a four-category exposure variable, and calculated the population attributable fraction ^28^ and E-values (appendix p 6). To investigate the effect of alternative handling of missing data we repeated the main analyses using multiple imputation with chained equations to replace missing data, imputing 50 datasets and combining using Rubin's rules.^29^ We used all variables from the analyses plus auxiliary variables (parental general health, and whether the young person had been truant from school or smoked). We required participants to have provided at least one GHQ-12 to improve the prediction of missing outcome data. We conducted two imputed analyses—both applying the survey weight from wave 1 to account for study design and non-response. First, we replaced missing data just in the outcome and confounders, and second, we replaced missing data in exposure, outcome, and confounders.

All analyses were conducted in Stata (version 16).

### Role of the funding source

The study was commissioned by the Department for Education. Study authors led all aspects of the study (design, analysis, and interpretation) in consultation with the Department for Education. Study authors were responsible for writing and submitting the paper.

## Results

The flow of participants through each cohort for each analysis is shown in the appendix (pp 7–10). Of 13 100 participants recruited to LSYPE2 in 2013, 6128 (46·8%) were included in the analysis of the association between higher education and symptoms of common mental disorders during higher education. 5045 (38·5%) of 13 100, 6732 (51·4%), 6753 (51·5%), and 6743 (51·5%) were included at ages 14–15 years, 16–17 years, 17–18 years, and 18–19 years, respectively. Of 16 122 recruited participants in LSYPE1 in 2004 and in the boost in 2007, 4832 (30·0%) were included in the analysis of associations between higher education and future symptoms of common mental disorders. Of the 16 122 participants, 7078 (43·9%), 8493 (52·7%) and 5611 (34·8%) were included at ages 14–15 years, 16–17 years, and 25 years, respectively.

Characteristics of the samples used in each cohort—overall and according to higher education attendance—are shown in table 1. In LSYPE2, 50·7% (3104 of 6128) were students in higher education, and 55·8% (2696 of 4832) were students in higher education in LSYPE1. There were differences in the characteristics of those who did and did not attend higher education—a higher proportion of higher education students had university-educated parents and came from higher socioeconomic backgrounds. Students from higher education were also less likely to have ever used cannabis or been bullied (table 1).

In LSYPE2, the raw mean GHQ-12 score during higher education was 12·0 (SD 6·4) for those who attended and 11·6 (SD 6·8) for those who did not attend higher education. In the univariable model, during higher education, GHQ-12 scores (adjusted mean difference) were 0·43 (95% CI 0·07 to 0·79; p=0·020) points higher in young people who attended higher education compared with those who did not attend (table 2). This attenuated after adjusting for sociodemographic factors (0·19, –0·16 to 0·55; p=0·29), but strengthened after additionally adjusting for antisocial behaviour, bullying, alcohol use, cannabis use, carer status, quality of health, and disability status (0·60, 0·26 to 0·94; p=0·0005) indicating negative confounding.^30^ The association remained (0·36, 0·05 to 0·68; p=0·024; table 2), after additionally adjusting for GHQ-12 scores at age 16–17 years. This final, fully adjusted estimate equated to a standardised mean difference of 0·06 and a population attributable fraction of 6%. The E-value was 1·3. There was no statistical evidence for an interaction by ethnicity (p=0·54) or parents’ education (p=0·22).

**Table 2:** Mean difference in symptoms of common mental disorders between individuals who did and did not attend higher education, before and after adjustments

	**Ages 18–19 years (LSYPE2; N=6128)**	**Age 25 years (LSYPE1; N=4832)**
Did not attend higher education	1-ref	1-ref
Model 1*	0·43 (0·07 to 0·79); p=0·020	−0·46 (−0·88 to −0·05); p=0·030
Model 2†	0·28 (−0·07 to 0·63); p=0·12	−0·51 (−0·93 to −0·09); p=0·018
Model 3‡	0·19 (−0·16 to 0·55); p=0·29	−0·31 (−0·73 to 0·12); p=0·15
Model 4§	0·32 (−0·03 to 0·67); p=0·074	−0·14 (−0·55 to 0·28); p=0·52
Model 5¶	0·39 (0·04 to 0·74); p=0·028	−0·08 (−0·50 to 0·34); p=0·72
Model 6‖	0·60 (0·26 to 0·94); p=0·0005	0·02 (−0·40 to 0·44); p=0·92
Model 7**	0·36 (0·05 to 0·68); p=0·024	−0·25 (−0·66 to 0·16); p=0·23

Data are mean difference (95% CI) with p values. A positive mean difference indicates a higher score in those who attend higher education. Data from main analysis complete case sample. Participants were included if they had complete data on all variables (exposure, outcome, and all study confounders). Analyses weighted using weight from main outcome timepoint, which is ages 18–19 years (wave 6) for LSYPE2 and age 25 years (wave 8) for LSYPE1.

* Unadjusted model.

† Adjusted for sex and ethnicity.

‡ Model 2 plus parents' socioeconomic status, parents' highest qualification, and family composition.

§ Model 3 plus antisocial behaviour and experienced bullying.

¶ Model 4 plus alcohol use and cannabis use.

‖ Model 5 plus carer status, general quality of health, and disability status.

** Model 6 plus GHQ-12 scores at previous timepoint—for LSYPE1, this is ages 16–17 years (wave 4) and for LSYPE2, this is ages 17–18 years (wave 5).

For LSYPE1, raw mean GHQ-12 scores after higher education were 11·4 (SD 5·5) for those who had attended higher education and 11·7 (SD 6·4) for those who had not attended. In the univariable model, GHQ-12 scores were 0·46 (95% CI –0·88 to –0·05; p=0·030) points lower in young people who had attended higher education compared with those who had not attended. The association attenuated after adjusting for confounders, with no association in the fully adjusted model (–0·25, –0·66 to 0·16; p=0·23; table 2). There was no statistical evidence for an interaction by ethnicity (p=0·77) or parents’ education (p=0·14).

The findings did not change when using the binary GHQ-12 (appendix p 12). Excluding individuals who took a gap year (appendix p 13) and imputing missing data (appendix pp 14–15) did not change the results. When using a four-category exposure, in LSYPE2, both higher education groups had higher GHQ-12 scores than those who were working or training (but not those who were not in education, employment, or training; appendix p 16).

There was evidence that associations between higher education attendance and symptoms of common mental disorders differed across timepoints in both studies (p=0·0015 in LSYPE2 and p=0·0023 in LSYPE1; figure; appendix p 19). In LSYPE2 at age 14–15 years, GHQ-12 scores were 0·38 (95% CI –0·74 to –0·01) points lower in young people who later attended higher education at age 18–19 years compared with those who did not. After adjustments the mean difference was larger at –0·74 (95% CI 1·09 to –0·39; table 3). At age 16–17 years, 17–18 years, and 18–19 years, GHQ-12 scores were slightly higher in those who attended higher education but all associations were attenuated in adjusted models (table 3). In LSYPE1 at age 14–15 years, GHQ-12 scores were 0·55 (95% CI 0·25 to 0·85) higher in young people who attended higher education at age 18–20 years than those who did not. But the association attenuated after adjustment for confounders (0·08, 95% CI –0·23 to 0·39; table 3). At age 16–17 years, those who attended higher education at ages 18–20 years had GHQ-12 scores 1·11 points higher (95% CI 0·82 to 1·40; table 3; figure) than young people who would not attend. After adjustment the score difference was 0·60 (95% CI 0·30 to 0·90; table 3). By the age of 25 years, there was no evidence of a difference between the two groups (figure).

**Figure:**
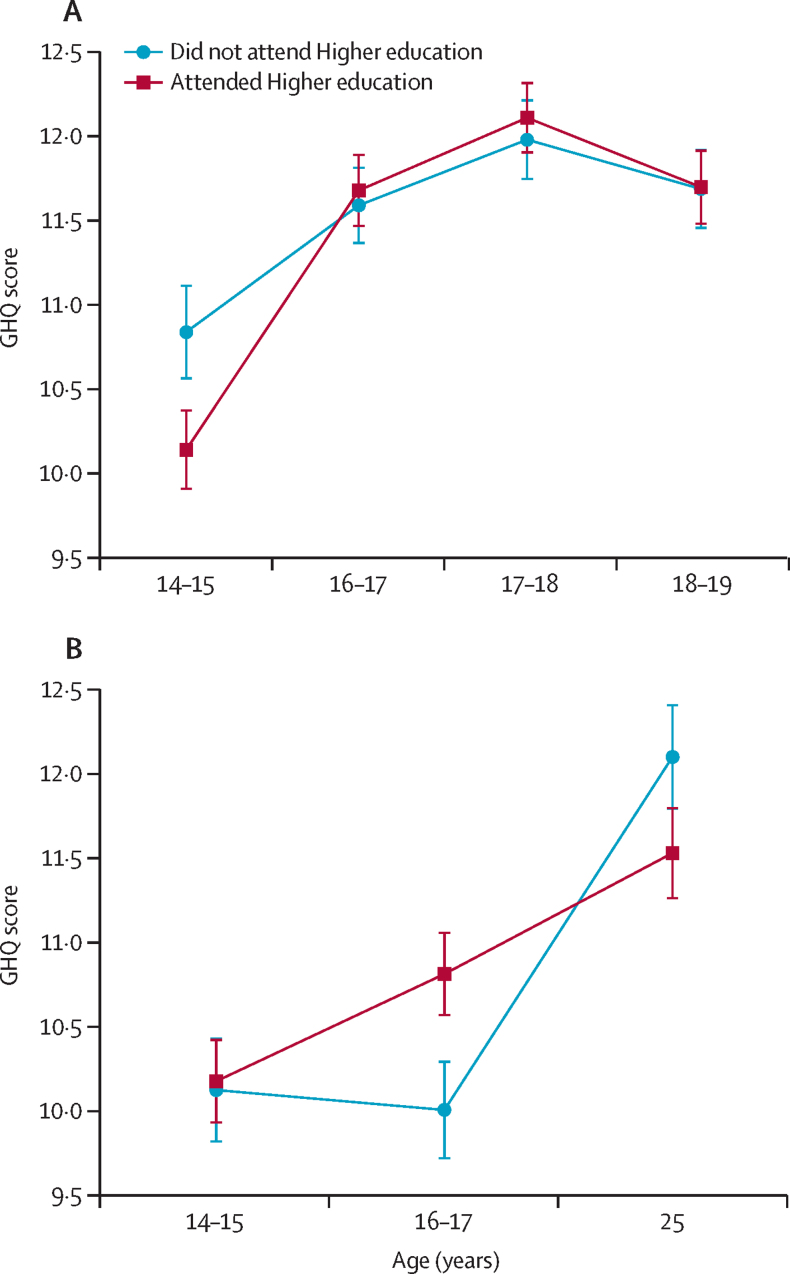
Mean (95% CIs) for GHQ-12 scores (symptoms of common mental disorders) over time in young people who attended higher education compared with those who did not attend for (A) LSYPE2 and (B) LSYPE1, from adjusted models GHQ=General Health Questionnaire. LSYPE1=Longitudinal Study of Young People in England. LSYPE2=Longitudinal Study of Young People in England cohort 2.

**Table 3:** Mean difference in symptoms of common mental disorders between young people who did and did not attend higher education, from multilevel model, separated by timepoint

	**LSYPE2**				**LSYPE1**		
	**Ages 14–15 years (wave 2; N=5045)**	**Ages 16–17 years (wave 4; N=6732)**	**Ages 17–18 years (wave 5; N=6753)**	**Ages 18–19 years (wave 6; N=6743)**	**Ages 14–15 years (wave 2; N=7078)**	**Ages 16–17 years (wave 4; N=8493)**	**Age 25 years (wave 8; N=5611)**
Did not attend higher education	1-ref	1-ref	1-ref	1-ref	1-ref	1-ref	1-ref
Model 1*	−0·38 (−0·74 to −0·01)	0·41 (0·09 to 0·72)	0·52 (0·20 to 0·85)	0·42 (0·07 to 0·77)	0·55 (0·25 to 0·85)	1·11 (0·82 to 1·40)	−0·34 (−0·75 to 0·07)
Model 2†	−0·74 (−1·09 to −0·39)	−0·06 (−0·38 to 0·25)	0·08 (−0·25 to 0·40)	0·16 (−0·18 to 0·50)	0·08 (−0·23 to 0·39)	0·60 (0·30 to 0·90)	−0·21 (−0·64 to 0·23)

Data are mean difference (95% CI). A positive mean difference indicates a higher score in those who attended higher education. Sample size differs by timepoint (as indicated). Participants were included if they had complete data on exposure, sex, ethnicity, and socioeconomic confounders, and at least one GHQ-12 at any timepoint. Each analysis is weighted using the weight from that timepoint.

* Model 1 is unadjusted.

† Model 2 is adjusted for sex, ethnicity, parents' socioeconomic status, parents' highest qualification, and family composition.

## Discussion

In this study, we found evidence that students who attended higher education in 2018 (LSYPE2) had a small increased risk of symptoms of common mental disorders while studying, compared with non-students. The effect size was small, which is reassuring for young people and their families, and suggests that higher education might not be a major contributor to common mental disorders among young people. We found evidence that, if the potential mental health risks of attending higher education were eliminated, the incidence of common mental disorders could possibly reduce by 6% (ie, the population attributable fraction if associations were causal). In the cohort born earlier, in 1989–90 (LSYPE1), we investigated common mental disorders at the age of 25 years and found no evidence of a difference between those who had attended higher education at ages 18–20 years and those who had not by this later timepoint. Students tend to come from higher parental socioeconomic status and parental education than non-students and so could be expected to have better mental health, consistent with evidence on the socioeconomic gradient of mental health. Hence, our finding of a small association in the opposite direction at age 18–19 years and no difference at age 25 years warrants further investigation. More research comparing students and non-students is needed to investigate the possibility that higher education environments could cause an increased risk of common mental disorders among young people.

We also compared differences in symptoms of common mental disorders before attending higher education (ie, during secondary school). In LSYPE1, we found that young people who would go on to attend higher education had more symptoms than those who did not, at age 16–17 years but not at age 14–15 years. In LSYPE2, we found that students who would go on to attend higher education had fewer symptoms of common mental disorders than non-students at ages 14–15 years, but not at any other timepoint during secondary school. These analyses did not show evidence of a difference between the two groups at age 18–19 years in LSYPE2. This lack of association in the secondary analysis might be due to the fewer confounders included in these models compared with the main analysis; only those that were measured before the earliest timepoint for the outcome (age 14–15 years) were included in the secondary analysis. We observed a strong pattern of negative confounding for the additional confounders used in the main analyses,^30^ supporting the suggestion that the backgrounds of students in higher education would usually lead to better mental health. It is unclear why the association between future higher education attendance and symptoms of common mental disorders during secondary school differed between cohorts. One possible explanation is a change in law between the cohorts that made post-16 education or training compulsory, therefore affecting the exposure to the final secondary school years. While at age 16–17 years in LSYPE1, young people who would not attend higher education would have already been allowed to leave education, in LSYPE2 they could not. This means that at age 16–17 years in LSYPE2, all participants were exposed to the potential stressors that come with being in education or training, whereas in LSYPE1, some, especially those not aiming for higher education, might have not been.

Our finding that, while being in higher education, students had more symptoms of common mental disorders than non-students was inconsistent with some previous studies.^14^, ^15^, ^17^ Most studies were conducted in different countries, used samples collected before 2011, and adjusted for fewer confounders. The most similar study to our study analysed data from individuals aged 17–24 years between 2010 and 2019 in the UK.^18^ In this study, young people who attended higher education had lower GHQ-12 scores than those who did not. However the association was only evident in overall pooled analyses and analyses stratified by year of data collection found no association in the most recent years, 2016–18 and 2017–19. Additionally, the confidence interval for the 2017–19 analysis overlapped with ours. Potential reasons to explain why this study did not detect the difference we did in 2017–19 include the broader age range of participants (17–24 years), the inclusion of participants from elsewhere in the UK, and adjustment for fewer confounders (given the strong pattern of negative confounding we observed). Furthermore, this study relied on cross-sectional data only, wherefore existing or prior psychological distress could influence the observed association (reverse causality).

Although timepoints for exposure and outcome measurement differed across cohorts in the present analysis, both can be considered prospective. In LSYPE1 follow-up was 6–7 years after young people started higher education. In LSYPE2, common symptoms of mental disorders were measured in May, after young people had started higher education the previous autumn. To further reduce the risk of potential for reverse causality we also adjusted for symptoms of common mental disorders before higher education.

There are several limitations that need to be considered in the interpretation of this study. Due to the study design it was not possible to conclude whether findings across cohorts differed because of age or cohort effects. It could be that higher education had a negative effect on young people's mental health in the LSYPE2 cohort, which was more recent, but not in LSYPE1 as a result of cohort effects. There have been reports of increases in academic pressure and an overall deterioration in mental health of young people in the time between cohorts.^7^ There was a large amount of missing data on the exposure variable due to non-response. There was also missing data at the age of 25 years in LSYPE1 due to attrition. Systematic missing data could have led to selection bias. However, we used survey weights, which should account for non-response and attrition, and increase representativeness. We also conducted sensitivity analyses using multiple imputation. Findings were unchanged, suggesting that missing data are unlikely to have affected our findings substantially. The self-reported GHQ-12 could lead to more measurement error than clinical interviews. However, we would expect measurement error to be random with respect to our hypotheses, and symptom measures capture variation in severity, increase statistical power, and reduce observer bias.^20^, ^31^ We did not have data on whether young people dropped out of higher education; however, if participants dropped out but still provided outcome data, this should not affect the validity of our findings. Although we found no evidence that findings differed according to ethnicity and parental education, there might have been insufficient statistical power to detect differences in these subgroup analyses. Although we adjusted for a wide range of confounders, residual confounding can rarely be ruled out in observational studies. Finally, given the differences in higher education conditions and the English setting, our findings might not be generalisable to other countries including other UK countries.

We found a small positive association between higher education and more symptoms of common mental disorders at age 18–19 years, but not at age 25 years. Our findings, and the mixed findings of previous research, highlights the need for more studies comparing the mental health of students in higher education with non-students including timepoints before, during, and after the age at which young people typically attend higher education, to investigate the possible effect of educational policies and practices on the mental wellbeing of young people.

## Data sharing

De-identified Longitudinal Studies of Young People in England (LSYPE1; sweeps 1–8) data are available from the UK Data Service: https://beta.ukdataservice.ac.uk/datacatalogue/studies/study?id=5545&type=Data%20catalogue&lt. Deidentified data from waves 1–8 of Longitudinal Studies of Young People in England cohort 2 (LSYPE2) are available for direct share through the Office for National Statistics (ONS) Secure Research Service: https://closer.ac.uk/study/lsype-2/. The use of the ONS statistical data in this work does not imply the endorsement of the ONS in relation to the interpretation or analysis of the statistical data.

For more on **Centre for Longitudinal Studies** see https://cls.ucl.ac.uk/cls-studies/next-steps

For more on **Cohort and Longitudinal Studies Enhancement Resources** see https://closer.ac.uk/study/lsype-2

## Declaration of interests

We declare no competing interests.
