# Supplementary material for: The association between higher education attendance and common mental health problems among young people in England: evidence from two population-based cohorts
Source: Lancet Public Health. 2023 Sep 28;8(10):e811–9. doi: 10.1016/S2468-2667(23)00188-3 (PMC10958987; doi:10.1016/S2468-2667(23)00188-3)
Supplement: Supplementary appendix 1 [file mmc1.pdf]

THE LANCET
Public Health

Supplementary appendix

This appendix formed part of the original submission and has been peer reviewed. We post it as supplied by the authors.

Supplement to: McCloud T, Kamenov S, Callender C, Lewis G, Lewis G. The association between higher education attendance and common mental health problems among young people in England: evidence from two population-based cohorts. *Lancet Public Health* 2023; **8:** e811–19.

Supplement Contents

Content	Page
Information on variables	3
Population attributable fraction (PAF) and E-values	6
Supplementary Figure 1: Sample flowchart in LSYPE2, for analyses of the association between higher education and current and future CMD symptoms.	7
Supplementary Figure 2: Sample flowchart in LSYPE2, for analyses of the association between higher education and CMD symptom trajectories.	8
Supplementary Figure 3: Sample flowchart in LSYPE1, for analyses of the association between higher education and current and future CMD symptoms.	9
Supplementary Figure 4: Sample flowchart in LSYPE1, for analyses of the association between higher education and CMD symptom trajectories.	10
Supplementary Table 1: LSYPE data and the availability of higher education and mental variables in each cohort.	11
Supplementary Table 2: Sensitivity analysis with common mental disorders outcome as a binary variable	12
Supplementary Table 3: Sensitivity analysis excluding those who took a gap year	13
Supplementary Table 4: Mean difference in symptoms of common mental disorders between young people who did and did not attend higher education, in sample with complete exposure data (missing data on outcome and confounders imputed)	14
Supplementary Table 5: Mean difference in symptoms of common mental disorders between young people who did and did not attend higher education, in sample with complete data (missing data on exposure, outcome and confounders imputed)	15
Supplementary Table 6: Mean difference in symptoms of common mental disorders at age 18/19 based on young person's main activity (four-category exposure variable), with higher education degree as the reference category.	16
Supplementary Table 7: Mean difference in symptoms of common mental disorders at age 18/19 based on young person's main activity (four-category exposure variable), with working/training as the reference category.	17
Supplementary Table 8: Mean difference in symptoms of common mental disorders at age 18/19 based on young person's main activity (four-category exposure variable), with other as the reference category.	18

Supplementary Table 9: Mean (SD) symptoms of common mental disorders at each time-point, in the sample overall and those who did and did not attend higher education	19
Supplementary Table 10: Demographic characteristics of included and excluded participants.	20

Information on variables

The following states how each included confounding or auxiliary variable was measured and when.

Sex

In LSYPE2, young person was judged by interviewer to be of male or female sex. In LSYPE1, young person was asked ‘are you male or female’, indicated as sex. This was measured at age 13/14 (wave 1) in LSYPE2 and age 18/19 (wave 6) in LSYPE1.

Ethnicity

In LSYPE2, young people were asked ‘What is your ethnic group?’ with 18 categories as response options. In LSYPE1, young people were asked ‘To which of the groups on this card would you say you belong?’ with 16 categories as response options. The two options missing from this version were “White – Gypsy or Irish Traveller” and “Arab”, instead categorised as “Any other White background” and “Other ethnic group”, respectively. The “Chinese” ethnic group was categorised within “Asian or Asian British” in LSYPE2 but within “Other ethnic group” in LSYPE1.

We used derived variables available in the datasets that grouped participants into eight ethnicity categories: White, Mixed, Indian, Pakistani, Bangladeshi, Black African, Black Caribbean, Other. This was measured at age 13/14 (wave 1) in LSYPE2 (with missing data supplemented with age 14/15 data) and age 16/17 (wave 4) in LSYPE1.

Parents’ Socioeconomic Status

Derived variables were used from the datasets that indicated which category each parent’s employment activity fell into from the following eight: Higher Managerial and professional occupations; Lower managerial and professional occupations; Intermediate occupations; Small employers and own account workers; Lower supervisory and technical occupations; Semi-routine occupations; Routine occupations; Not currently working. These categories are based on the NS-SEC operational category tool for socioeconomic class, which has 41 categories. Categories were collapsed due to small numbers in some groups, resulting in the following three categories: Managerial and professional occupations, Intermediate occupations, Lower supervisory, routine occupations and not currently working. We then combined the two parents’ variables into one variable that indicated the employment category of whichever parent had the highest value. This was measured at age 13/14 (wave 1) in LSYPE2 and age 16/17 (wave 4) in LSYPE1.

Parents’ Highest Qualification

This indicates the qualification held by whichever parent has the highest qualification. In LSYPE2, this is either the mother or father, and in LSYPE1, this is either the main or second parent. We used derived variables available in the datasets that indicated the highest qualification held out of seven categories: Degree or equivalent; Higher education below degree level; GCE A Level or equivalent; GCSE grades A-C or equivalent; Qualifications at level 1 and below; Other qualifications; No qualifications. These categories are based on a detailed list of 50 qualifications. In LSYPE1, interviewers collected data pertaining to the 50 categories. In LSYPE2, only the seven-category answer was recorded. Categories were collapsed due to small numbers in some groups, resulting in the following five categories:

Degree or equivalent, Higher education below degree level, GCE, A Level or equivalent, GCSE grades A-C or equivalent, Below GCSE or no qualification. This was measured at age 13/14 (wave 1) in LSYPE2 and age 16/17 (wave 4) in LSYPE1.

Family Composition
This indicates the family level composition based on the (natural, step, adoptive or foster) parents of the young person. We used derived variables available in the datasets that were based on questions asked to parents, indicating one of the following five situations: Married couple; Cohabiting couple; Lone father; Lone mother; No parents in the household. In the derived variables, if there is only one parent (mother or father) in the household, the family is coded as a lone father/mother. If there is one mother and one father in the household, then relationship variables are used to determine whether the couple are married or cohabiting. If there are two mothers or fathers in the household, these are assumed to be same sex couples coded as cohabiting. Categories were collapsed due to small numbers in some groups, resulting in two categories: Married/cohabiting or Lone parent or no parents in the household. This was measured at age 13/14 (wave 1) in LSYPE2 and age 16/17 (wave 4) in LSYPE1.

Antisocial Behaviour
This indicates whether the young person has taken part in antisocial behaviour in the previous 12 months. In LSYPE2, antisocial behaviour includes the following: damaging anything in a public place on purpose that does not belong to them; shoplifting; graffitiing anywhere; hitting or attacking someone on purpose with or without using an object or weapon. In LSYPE1, it includes the following: vandalising public property; shoplifting; graffitiing on walls; fighting or public disturbance. This was measured at age 15/16 (wave 3) in both LSYPE2 and LSYPE1.

Experienced Bullying
This indicates whether the young person has been bullied in any way in the previous 12 months. In LSYPE2, this includes being upset by name-calling (including by text or email), being excluded from a group of friends, being made to hand over money or possessions, being threatened with violence by other students, experiencing violence from other students, and being bothered, harassed or having hurtful words, pictures or videos spread about them via internet or mobile phone. In LSYPE1, this includes all of the above except the latter item about cyber-bullying. This was measured at age 15/16 (wave 3) in LSYPE2 and LSYPE1.

Frequency of Alcohol Use
This was measured at age 16/17 (wave 4) in LSYPE2 and LSYPE1. There were two questions used to create this variable. Young people were asked:
1. Have you ever had a proper alcoholic drink? That is a whole drink, not just a sip. Please do not count drinks labelled low alcohol.
If they answered yes, they were then asked the second question:
2. Thinking about the last 12 months, about how often did you usually have an alcoholic drink?

We combined the two questions so that those who answered no to the first question were coded as 'Never' in response to the second question. The response options given for the second question were slightly different in each cohort, so we recoded them to be more comparable. In LSYPE2, the categories for the second question were as follows: 4+ times a week, 2-3 times a week, 2-3 times a month, Once a month or less, Never (given as an option for the second question, as well as containing those who answered no to the first question). We collapsed the former two categories. In LSYPE1, the categories for the second question were as follows: Most days, Once or twice a week, 2 or 3 times a month, Once a month, Once every couple of months, Less often, Never (not given as an option for the second question, so only contains those who answered no to the first question). We collapsed the former two categories, then the following two, then the following two. The final combined categories were therefore as follows: Never, Less than monthly, A few times a month, Weekly or more.

Cannabis Use
Young people were asked whether they had ever tried cannabis, even if only once. This was measured at age 16/17 (wave 4) in LSYPE2 and LSYPE1.

General Quality of Health
Young people were asked 'In the last 12 months, would you say your health has been very good, fairly good, not very good, or not good at all?' The latter two categories were combined. This was measured at age 16/17 (wave 4) in LSYPE2 and LSYPE1.

Disability Status
Indicates whether the young person has any longstanding illness, disability or infirmity. This was a question of opinion, asked to young people with the added instruction 'By 'longstanding' I mean anything that has troubled you over a period of at least 12 months or that is likely to affect you over a period of at least 12 months?'. This was measured at age 16/17 (wave 4) in LSYPE2 and LSYPE1.

Carer Status
In LSYPE2, this indicates whether young person has been a carer at age 16/17 (wave 4) only. In LSYPE1, indicates whether young person has been a carer at age 16/17 (wave 4) or age 17/18 (wave 5). Young people were asked 'Do you regularly look after any ill, disabled or elderly relatives or friends aged 15 or more and in need of care, without being paid? This includes both people who live here with you and those who live elsewhere.' This was clarified as not including any professional obligations such as volunteering.

Parent General Health
In LSYPE1, the young person's main parent was asked 'Do you have any longstanding illness, disability, or infirmity?' The response options were yes or no. In LSYPE2, the young person's mother was asked 'In the last 12 months, would you say your health has been very good, fairly good, not very good, or not good at all?' The latter two categories were combined. This was measured at age 13/14 (wave 1) in LSYPE2 and LSYPE1.

Truancy

This indicates whether young people have missed school without permission in the last 12 months. Young people were asked ‘In the last 12 months, have you ever played truant, that is missed school without permission, even if it was only for a half day or a single lesson?’ This was measured at age 13/14 (wave 1) in LSYPE2 and LSYPE1.

Smoking

Young people were asked whether they ever smoke cigarettes at all. Those who indicated that they did were then asked to indicate the statement that best describes them out of the following: I have never smoked; I have only ever tried smoking once; I used to smoke sometimes but I never smoke a cigarette now; I sometimes smoke cigarettes now but I don't smoke as many as one a week; I usually smoke between one and six cigarettes a week; I usually smoke more than six cigarettes a week. These two questions were combined so that those who answered no to the first question were coded as ‘Never smoked’ in response to the second question. The categories were then collapsed to the following: Never smoked; Sometimes or less often; One or more per week. This was measured at age 13/14 (wave 1) in LSYPE2 and LSYPE1.

Population attributable fraction and E-values

The PAF represents the reduction in incidence that would occur if the risks associated with the exposure were eliminated, based on the assumptions that the exposure is causal and the estimate valid[1]. We found evidence that, in LSYPE2, the PAF was 6% for the association between higher education attendance and CMD symptoms. Given the high prevalence of CMD, their rising incidence rates among young people, and how little we know about prevention, we think this finding is important and valuable to public health.

The E-value is an approach to sensitivity analyses for unmeasured confounding in observational studies. The E-value quantifies the minimum strength of association that an unmeasured confounder must have with both exposure and outcome, while simultaneously considering the measured covariates, to negate the observed association. If the strength of unmeasured confounding is weaker than indicated by the E-value, then the main study result could not be overturned to one of “no association”[2]. The E-value for the association between higher education attendance and CMD symptoms in LSYPE2 was 1.3. In our study, most confounders were increasing rather than decreasing the size of the association (negative confounding). If unmeasured confounders also have this effect, it would strengthen rather than weaken our association. Furthermore, the E-value of 1.3 is larger the point estimate for any confounder we adjusted for. So, in this context, the E-value can be considered as relatively large (because unmeasured confounding would have to have larger effects than most measured confounders to account for the association).

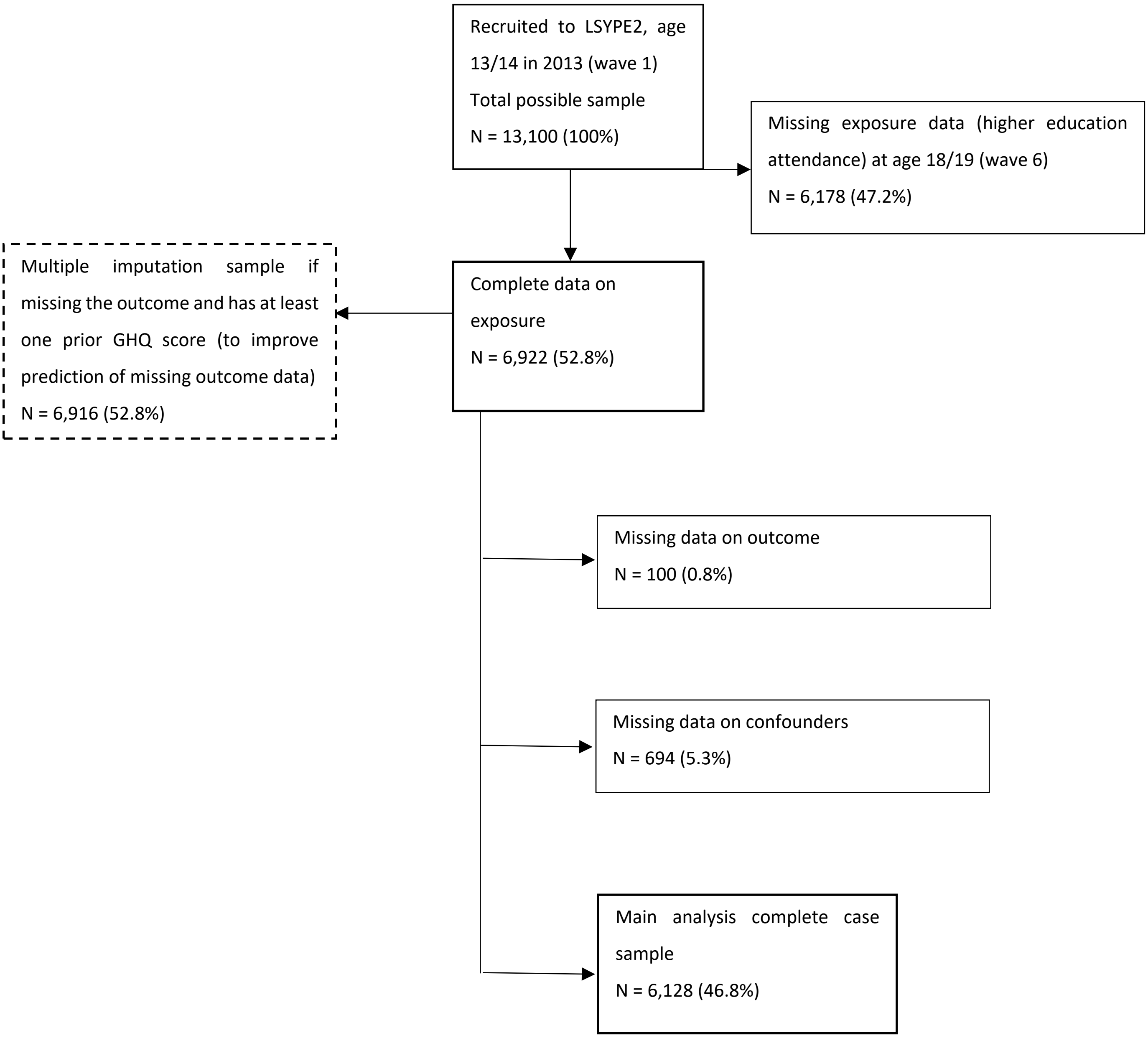

Supplementary Figure 1: Sample flowchart in LSYPE2, for analyses of the association between higher education and current and future CMD symptoms. Flowchart begins with the sample initially recruited at wave 1.

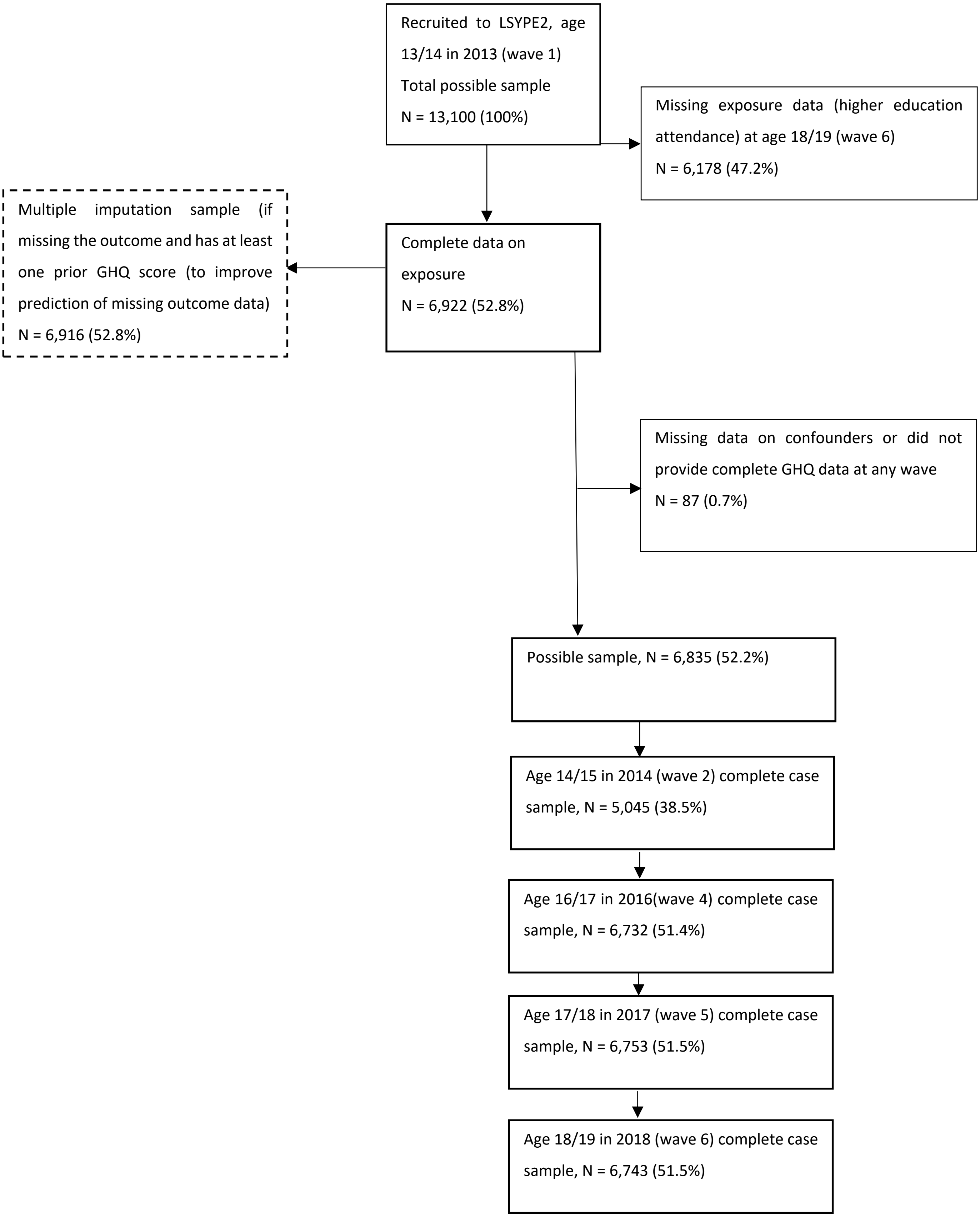

Supplementary Figure 2: Sample flowchart in LSYPE2, for analyses of the association between higher education and CMD symptom trajectories. Flowchart begins with the sample initially recruited at wave 1.

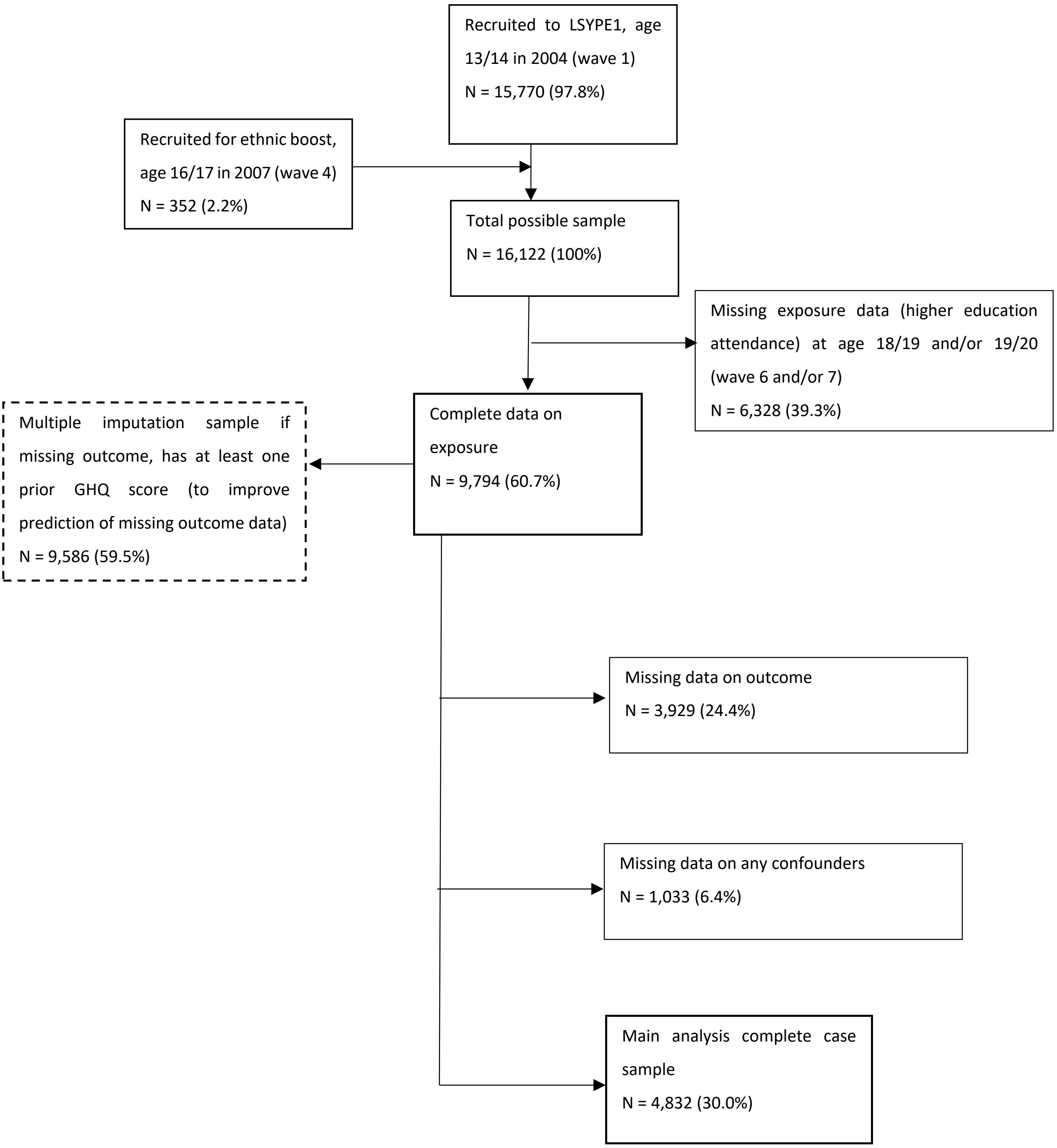

Supplementary Figure 3: Sample flowchart in LSYPE1, for analyses of the association between higher education and current and future CMD symptoms. Flowchart begins with the sample initially recruited at wave 1.

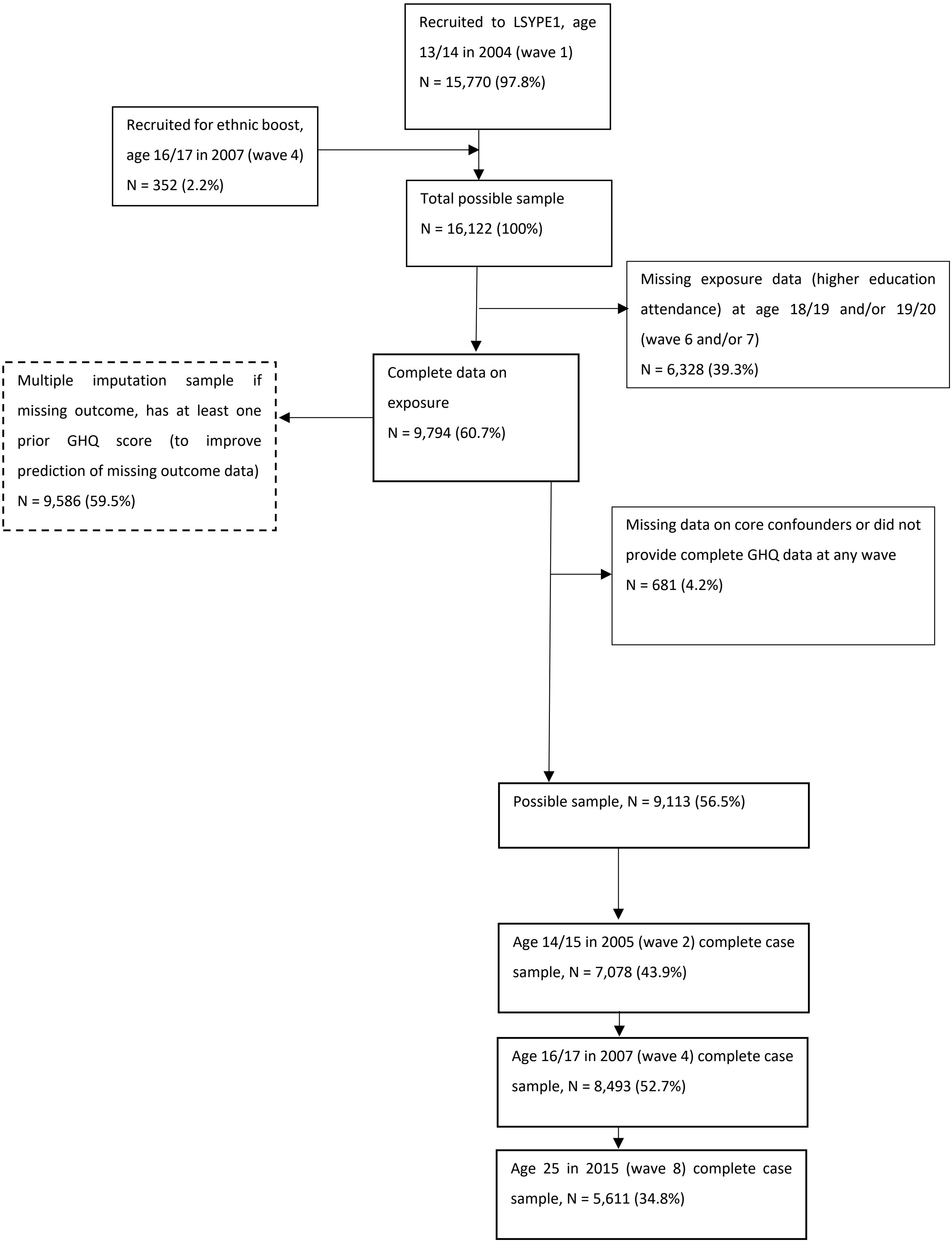

Supplementary Figure 4: Sample flowchart in LSYPE1, for analyses of the association between higher education and CMD symptom trajectories. Flowchart begins with the sample initially recruited at wave 1.

Supplementary Table 1: LSYPE data and the availability of higher education and mental variables in each cohort.

Data collection					Variable			
					Higher education		Common mental disorder symptoms (GHQ-12)	
Wave	Age	School year (UK)	Calendar year		LSYPE1	LSYPE2	LSYPE1	LSYPE2
			LSYPE1	LSYPE2				
1	13/14	9	2004	2013	No	No	No	No
2	14/15	10	2005	2014	No	No	Yes	Yes
3	15/16	11	2006	2015	No	No	No	No
4	16/17	12	2007	2016	No	No	Yes	Yes
5	17/18	13	2008	2017	No	No	No	Yes
6	18/19	-	2009	2018	Yes[a]	Yes[a]	No	Yes
7	19/20	-	2010	2019	Yes[a]	N/A[b]	No	N/A[d]
8	25	-	2015	-	Yes[a]	N/A[b]	Yes	N/A[d]

[a]Data on whether the young person is currently studying for a higher education degree.

[b] Wave 7 and 8 data from LSYPE2 not available at time of analysis.

GHQ-12 = General Health Questionnaire.

Supplementary Table 2: Sensitivity analysis with common mental disorders outcome as a binary variable.

In LSYPE2, 33% (n = 1,035) of those who attended higher education and 30% (n = 916) of those who did not attend exceeded the threshold for CMD. In LSYPE1, these figures were 30% (n = 812) of those who attended and 30% (n = 640) of those who did not attend, respectively.

**Model**	**Odds Ratio (95% Confidence Interval), p value**	
	**Age 18/19 (LSYPE2)<br>N = 6,128**	**Age 25 (LSYPE1)<br>N = 4,832**
**Did not attend higher education**	*Reference category*	*Reference category*
**Model 1[a]**	1.17 (1.03 to 1.32), p = .013	0.95 (0.82 to 1.11), p = .51
**Model 2[b]**	1.11 (0.98 to 1.26), p = .11	0.93 (0.80 to 1.08), p = .32
**Model 3[c]**	1.08 (0.95 to 1.22), p = .25	1.00 (0.85 to 1.17), p = .96
**Model 4[d]**	1.13 (0.99 to 1.28), p = .064	1.06 (0.90 to 1.25), p = .49
**Model 5[e]**	1.15 (1.02 to 1.31), p = .027	1.08 (0.92 to 1.28), p = .36
**Model 6[f]**	1.22 (1.08 to 1.39), p = .0022	1.11 (0.94 to 1.31), p = .22
**Model 7[g]**	1.17 (1.02 to 1.34), p = .024	1.03 (0.87 to 1.22), p = .74

a. Unadjusted model.
b. Adjusted for sex and ethnicity.
c. Model 2 plus parents' socioeconomic status, parents' highest qualification and family composition.
d. Model 3 plus antisocial behaviour and experienced bullying.
e. Model 4 plus alcohol use and cannabis use.
f. Model 5 plus carer status, general quality of health and disability status.
g. Model 6 plus GHQ-12 scores at previous wave - for LSYPE2, this is age 16/17 (wave 4) and for LSYPE1, this is age 17/18 (wave 5).

Notes:
1. Data from Analysis 1 complete case sample - N = 6,128 for LSYPE2 and N = 4,832 for LSYPE1.
2. Analyses weighted using weight from primary outcome wave – age 18/19 (wave 6) for LSYPE2 and age 25 (wave 8) for LSYPE1.
3. GHQ scores made binary by coding each item 0 or 1 - a score of 1 or 2 would be coded 0 and a score of 3 or 4 would be coded 1. The score across the 12 items is then totalled. Finally, any participant with a total score above 2 would be coded as 1, and scores of 2 and below would be coded as 0.

Supplementary Table 3: Sensitivity analysis excluding those who took a gap year.

Model	Mean Difference (95% Confidence Interval), p value
	**Age 25 (LSYPE1)**<br>**N = 4,824**
Did not attend higher education	*Reference category*
Model 1[a]	-0.44 (-0.84 to -0.03), p = .033
Model 2[b]	-0.49 (-0.89 to -0.09), p = .017
Model 3[c]	-0.30 (-0.70 to 0.09), p = .13
Model 4[d]	-0.16 (-0.56 to 0.24), p = .44
Model 5[e]	-0.08 (-0.48 to 0.33), p = .71
Model 6[f]	0.04 (-0.36 to 0.44), p = .84
Model 7[g]	-0.14 (-0.54 to 0.25), p = .48

a. Unadjusted model.
b. Adjusted for sex and ethnicity.
c. Model 2 plus parents' socioeconomic status, parents' highest qualification and family composition.
d. Model 3 plus antisocial behaviour and experienced bullying.
e. Model 4 plus alcohol use and cannabis use.
f. Model 5 plus carer status, general quality of health and disability status.
g. Model 6 plus GHQ-12 scores at previous wave - for LSYPE1, this is age 17/18 (wave 5).

Notes:

1. Categorises higher education variable using only data from age 18/19 (wave 6), comparable with the data available in LSYPE2.
2. Uses alternate Analysis 1 complete case sample – those with complete data on primary outcome, all confounders and exposure (now only coded using data from wave 6). N = 4,824.
3. Analyses weighted using weight from primary outcome wave – age 25 (wave 8) for LSYPE1.

Supplementary Table 4: Mean difference in symptoms of common mental disorders between young people who did and did not attend higher education, in sample with complete exposure data (missing data on outcome and confounders imputed).

**Model**	**Mean Difference (95% Confidence Interval), p value**	
	**Age 18/19 (LSYPE2) N = 6,916**	**Age 25 (LSYPE1) N = 9,586**
Did not attend higher education	*Reference category*	*Reference category*
Model 1[a]	0.46 (0.19 to 0.82) p = .011	-0.25 (-0.59 to 0.09) p = .14
Model 2[b]	0.29 (-0.05 to 0.63) p = .096	-0.31 (-0.65 to 0.03) p = .071
Model 3[c]	0.22 (-0.13 to 0.57) p = .22	-0.13 (-0.50 to 0.24) p = .50
Model 4[d]	0.33 (-0.01 to 0.67) p = .058	0.02 (-0.35 to 0.39) p = .91
Model 5[e]	0.39 (0.05 to 0.73) p = .026	0.07 (-0.03 to 0.44) p = .72
Model 6[f]	0.62 (0.29 to 0.95) p < .001	0.17 (-0.20 to 0.54) p = .36
Model 7[g]	0.39 (0.09 to 0.70) p = .011	-0.15 (-0.51 to 0.22) p = .43

a. Unadjusted model.
b. Adjusted for sex and ethnicity.
c. Model 2 plus parents' socioeconomic status, parents' highest qualification and family composition.
d. Model 3 plus antisocial behaviour and experienced bullying.
e. Model 4 plus alcohol use and cannabis use.
f. Model 5 plus carer status, general quality of health and disability status.
g. Model 6 plus GHQ-12 scores at previous wave - for LSYPE2, this is age 16/17 (wave 4) and for LSYPE1, this is age 17/18 (wave 5).

Notes:
1. N = 6,916 for LSYPE2 and N = 9,586 for LSYPE1.
2. Analyses weighted to represent the target population using the sample weight from wave 1.

Supplementary Table 5: Mean difference in symptoms of common mental disorders between young people who did and did not attend higher education, in sample with complete data (missing data on exposure, outcome and confounders imputed).

**Model**	**Mean Difference (95% Confidence Interval), p value**	
	Age 18/19 (LSYPE2)<br>N = 13,100	Age 25 (LSYPE1)<br>N = 15,770
Did not attend higher education	*Reference category*	*Reference category*
Model 1[a]	0.41 (0.06 to 0.75) p = .020	-0.31 (-0.64 to 0.03) p = .073
Model 2[b]	0.24 (-0.10 to 0.58) p = .17	-0.36 (-0.69 to -0.02) p = .037
Model 3[c]	0.17 (-0.18 to 0.53) p = .34	-0.15 (-0.52 to 0.22) p = .41
Model 4[d]	0.29 (-0.06 to 0.64) p = .10	0.00 (-0.37 to 0.38) p = .99
Model 5[e]	0.35 (0.00 to 0.61) p = .049	.056 (-0.33 to 0.44) p = .77
Model 6[f]	0.59 (0.25 to 0.93) p = .001	0.16 (-0.22 to 0.55) p = .40
Model 7[g]	0.35 (0.05 to 0.65) p = .024	-0.17 (-0.54 to 0.20) p = .36

a. Unadjusted model.
b. Adjusted for sex and ethnicity.
c. Model 2 plus parents' socioeconomic status, parents' highest qualification and family composition.
d. Model 3 plus antisocial behaviour and experienced bullying.
e. Model 4 plus alcohol use and cannabis use.
f. Model 5 plus carer status, general quality of health and disability status.
g. Model 6 plus GHQ-12 scores at previous wave - for LSYPE2, this is age 16/17 (wave 4), for LSYPE1, this is age 17/18 (wave 5).

Notes:
1. N = 13,100 for LSYPE2 and N = 15,770 for LSYPE1.
2. Analyses weighted to represent the target population using the sample weight from wave 1.

Supplementary Table 6: Mean difference in symptoms of common mental disorders at age 18/19 based on young person's main activity (four-category exposure variable), with higher education degree as the reference category.

Exposure	LSYPE2				LSYPE1			
	**N (%)**	**Mean (SD)**	**Mean difference (95% CI)**	**P value**	**N (%)**	**Mean (SD)**	**Mean difference (95% CI)**	**P value**
Higher education degree[a]	2,509 (40.9%)	12.13 (6.40)	*Reference category*	*Reference category*	2,341 (48.5%)	11.37 (5.44)	*Reference category*	*Reference category*
Higher education other[b]	595 (9.7%)	11.49 (6.29)	-0.25 (-0.74 to 0.24)	.31	242 (5.0%)	11.43 (6.02)	0.27 (-0.56 to 1.09)	.52
Working/training[c]	2,299 (37.5%)	11.21 (6.45)	-0.62 (-0.97 to -0.28)	0004	1,865 (38.6%)	11.48 (11.21)	-0.03 (-0.46 to 0.39)	.87
NEET[d]	725 (11.8%)	12.95 (7.78)	0.19 (-0.37 to 0.74)	.51	384 (8.0%)	13.14 (7.62)	2.02 (1.06 to 2.98)	<.0001

a. Studying for a higher education degree
b. Studying in higher education but for a qualification other than degree (e.g. teacher training, higher apprenticeship, diploma)
c. Working/training (i.e. not in higher education but in another form of education, employment, or training)
d. Not in Education, Employment, or Training

Notes:
1. Data from Analysis 1 complete case sample - N = 6,128 for LSYPE2 and N = 4,832 for LSYPE1.
2. Data from fully adjusted model from main analysis (Model 7).
3. Analyses weighted using weight from primary outcome wave – age 18/19 (wave 6) for LSYPE2 and age 25 (wave 8) for LSYPE1.

Supplementary Table 7: Mean difference in symptoms of common mental disorders at age 18/19 based on young person's main activity (four-category exposure variable), with working/training as the reference category.

Exposure	LSYPE2				LSYPE1			
	**N (%)**	**Mean (SD)**	**Mean difference (95% CI)**	**P value**	**N (%)**	**Mean (SD)**	**Mean difference (95% CI)**	**P value**
Working/training[a]	2,299 (37.5%)	11.21 (6.45)	*Reference category*	*Reference category*	1,865 (38.6%)	11.48 (11.21)	*Reference category*	*Reference category*
Higher education degree[b]	2,509 (40.9%)	12.13 (6.40)	0.62 (0.28 to 0.97)	0004	2,341 (48.5%)	11.37 (5.44)	0.03 (-0.39 to 0.46)	.87
Higher education other[c]	595 (9.7%)	11.49 (6.29)	0.37 (-0.13 to 0.87)	.14	242 (5.0%)	11.43 (6.02)	0.30 (-0.52 to 1.12)	.47
NEET[d]	725 (11.8%)	12.95 (7.78)	0.81 (0.28 to 1.34)	.0027	384 (8.0%)	13.14 (7.62)	2.05 (1.08 to 3.03)	<.0001

a. Working/training (i.e. not in higher education but in another form of education, employment, or training)
b. Studying for a higher education degree
c. Studying in higher education but for a qualification other than degree (e.g. teacher training, higher apprenticeship, diploma)
d. Not in Education, Employment, or Training

Notes:
1. Data from Analysis 1 complete case sample - N = 6,128 for LSYPE2 and N = 4,832 for LSYPE1.
2. Data from fully adjusted model from main analysis (Model 7).
3. Analyses weighted using weight from primary outcome wave – age 18/19 (wave 6) for LSYPE2 and age 25 (wave 8) for LSYPE1.

Supplementary Table 8: Mean difference in symptoms of common mental disorders at age 18/19 based on young person's main activity (four-category exposure variable), with other as the reference category.

Exposure	LSYPE2				LSYPE1			
	**N (%)**	**Mean (SD)**	**Mean difference (95% CI)**	**P value**	**N (%)**	**Mean (SD)**	**Mean difference (95% CI)**	**P value**
NEET[a]	725 (11.8%)	12.95 (7.78)	*Reference category*	*Reference category*	384 (8.0%)	13.14 (7.62)	*Reference category*	*Reference category*
Higher education degree[b]	2,509 (40.9%)	12.13 (6.40)	-0.19 (-0.74 to 0.37)	.51	2,341 (48.5%)	11.37 (5.44)	-2.02 (-2.98 to -1.06)	<.0001
Higher education other[c]	595 (9.7%)	11.49 (6.29)	-0.44 (-1.08 to 0.20)	.18	242 (5.0%)	11.43 (6.02)	-1.75 (-2.95 to 0.56)	.0041
Working/training[d]	2,299 (37.5%)	11.21 (6.45)	-0.81 (-1.34 to -0.28)	.0027	1,865 (38.6%)	11.48 (11.21)	-2.05 (-3.03 to -1.08)	<.0001

a. Not in Education, Employment, or Training
b. Studying for a higher education degree
c. Studying in higher education but for a qualification other than degree (e.g. teacher training, higher apprenticeship, diploma)
d. Working/training (i.e. not in higher education but in another form of education, employment, or training)

Notes:
1. Data from Analysis 1 complete case sample - N = 6,128 for LSYPE2 and N = 4,832 for LSYPE1.
2. Data from fully adjusted model from main analysis (Model 7).
3. Analyses weighted using weight from primary outcome wave – age 18/19 (wave 6) for LSYPE2 and age 25 (wave 8) for LSYPE1.

Supplementary Table 9: Mean (SD) symptoms of common mental disorders at each time-point, overall and those who did and did not attend higher education

	Mean (95% Confidence Interval); standard deviation							
**Time point**	**LSYPE2**				**LSYPE1**			
	**Attended higher education**	**Did not attend higher education**	**Total**	**N**	**Attended higher education**	**Did not attend higher education**	**Total**	**N**
Age 14/15 (wave 2)	10.31 (10.08 to 10.54); 6.69	10.74 (10.48 to 11.01); 6.07	10.52 (10.34 to 10.69); 6.38	5,045	10.09 (9.92 to 10.27); 5.36	9.65 (9.45 to 9.85); 5.88	9.89 (9.76 to 10.02); 5.61	7,078
Age 16/17 (wave 4)	12.01 (11.80 to 12.22); 6.12	11.70 (11.48 to 11.92); 6.52	11.85 (11.70 to 12.01); 6.33	6,732	10.85 (10.68 to 11.03); 5.81	9.77 (9.59 to 9.95); 5.92	10.32 (10.19 to 10.44); 5.89	8,493
Age 17/18 (wave 5)	12.49 (12.28 to 12.70); 6.15	11.99 (11.77 to 12.21); 6.69	12.24 (12.08 to 12.39); 6.43	6,753				
Age 18/19 (wave 6)	12.07 (11.85 to 12.29); 6.41	11.73 (11.50 to 11.96); 6.84	11.90 (11.74 to 12.06); 6.63	6,743				
Age 25 (wave 8)					11.51 (11.31 to 11.71); 5.64	11.81 (11.56 to 12.06); 6.42	11.65 (11.49 to 11.80); 6.00	5,611

Notes:
1. Data from sample used for analyses. Participants were eligible to be included if they had complete data on exposure, core confounders, and at least one GHQ-12 at any time point. Sample size differs by time point (as indicated) based on when participants had provided GHQ-12 data.
2. Data are unweighted.
3. Grey cells indicate time points where data was not available for that cohort.

Supplementary Table 10: Demographic characteristics of included and excluded participants.

**Variable** – N (%)	**LSYPE2**		**LSYPE1**	
	**Included sample (n = 6,128)**	**Excluded sample (n = 6,972)**	**Included sample (n = 4,832)**	**Excluded sample (n = 11,290)**
**Sex**[b]				
Female	3,240 (52.9%)	3,093 (44.4%)	2,614 (54.1%)	2,257 (45.5%)
Male	2,888 (47.1%)	3,879 (55.6%)	2,218 (45.9%)	2,705 (54.5%)
**Ethnicity**[c]				
White	4,752 (77.6%)	5,120 (75.2%)	3,554 (73.6%)	3,086 (62.6%)
Mixed	260 (4.2%)	303 (4.5%)	207 (4.3%)	265 (5.4%)
Indian	159 (2.6%)	137 (2.0%)	332 (6.9%)	356 (7.2%)
Pakistani	221 (3.6%)	237 (3.5%)	246 (5.1%)	313 (6.3%)
Bangladeshi	160 (2.6%)	167 (2.5%)	188 (3.9%)	250 (5.1%)
Black African	251 (4.1%)	363 (5.3%)	97 (2.0%)	269 (5.5%)
Black Caribbean	166 (2.7%)	267 (3.9%)	103 (2.1%)	256 (5.2%)
Other	159 (2.6%)	212 (3.1%)	105 (2.2%)	138 (2.8%)
**Parents' Socioeconomic Status**[d,e]				
Managerial and professional occupations	2,933 (47.9%)	2,228 (32.9%)	2,340 (48.4%)	1,813 (40.1%)
Intermediate occupations	1,414 (23.1%)	1,558 (23.0%)	927 (19.2%)	798 (17.7%)
Lower supervisory, routine occupations and not currently working	1,781 (29.1%)	2,996 (44.2%)	1,565 (32.4%)	1,910 (42.4%)
**Parents' Highest Qualification**[e, f]				
Degree or equivalent	1,005 (16.4%)	719 (10.6%)	1,039 (21.5%)	812 (17.1%)
Higher education below degree level	718 (11.7%)	632 (9.3%)	855 (17.7%)	698 (14.7%)
GCE, A Level or equivalent	766 (12.5%)	724 (10.6%)	864 (17.9%)	778 (16.4%)
GCSE grades A-C or equivalent	2,555 (41.7%)	2,956 (43.4%)	1,131 (23.4%)	1,130 (23.8%)
Below GCSE or no qualification	1,084 (17.7%)	1,783 (26.2%)	943 (19.5%)	1,320 (27.9%)
**Family Composition**[e]				
Married/cohabiting	4,625 (75.5%)	4,345 (62.8%)	3,782 (78.3%)	3,289 (67.8%)
Lone parent or no parents in the household	1,503 (24.5%)	2,569 (37.2%)	1,050 (21.7%)	1,559 (32.2%)
**Antisocial Behaviour** (in past 12 months)[g, h]	469 (7.7%)	405 (11.9%)	764 (15.8%)	960 (21.0%)
**Experienced Bullying** (in past 12 months)[h]	1,809 (29.5%)	979 (31.9%)	1,287 (26.6%)	1,149 (26.7%)
**Frequency of Alcohol Use**[i, j]				

Never	1,987 (32.4%)	952 (35.0%)	1,033 (21.4%)	1,238 (27.2%)
Once every couple of months or less	2,486 (40.6%)	1,060 (38.9%)	865 (17.9%)	792 (17.4%)
1-3 times a month	1,400 (22.9%)	566 (20.8%)	1,529 (31.6%)	1,183 (26.0%)
Once a week or more	255 (4.2%)	144 (5.3%)	1,405 (29.1%)	1,341 (29.4%)
**Cannabis Use**[j] (ever)	1,325 (21.6%)	729 (26.5%)	1,458 (30.2%)	1,586 (34.1%)
**General Quality of Health**[j]				
Very good	2,490 (40.6%)	1,116 (40.2%)	2,519 (52.1%)	2,418 (51.0%)
Fairly good	3,103 (50.6%)	1,385 (49.8%)	1,992 (41.2%)	1,979 (41.7%)
Not very good or not good at all	535 (8.7%)	278 (10.0%)	321 (6.6%)	347 (7.3%)
**Disability Status**[j]	698 (11.4%)	365 (13.3%)	340 (7.0%)	359 (7.4%)
**Carer Status**[k]	305 (5.0%)	155 (5.5%)	368 (7.6%)	473 (9.7%)

a. Indicates whether young person was attending higher education at age 18/19 (wave 6) in LSYPE2, or at age 18/19 or 19/20 (wave 6 or 7) in LSYPE1.
b. Measured at age 13/14 (wave 1) in LSYPE2 and age 18/19 (wave 6) in LSYPE1.
c. Measured at age 13/14 (wave 1) in LSYPE2 (missing data supplemented with age 14/15 data) and age 16/17 (wave 4) in LSYPE1.
d. Parents' socioeconomic status is based on the socioeconomic status of whichever parent (mother or father) has the highest employment category.
e. Measured at age 13/14 (wave 1) in LSYPE2 and age 16/17 (wave 4) in LSYPE1.
f. Indicates the qualification held by whichever parent has the highest qualification. In LSYPE2, the mother or father, and in LSYPE1, the main or second parent.
g. In LSYPE2, antisocial behaviour includes taking part in any of the following: damaging anything in a public place on purpose that does not belong to them; shoplifting; graffitiing anywhere; hitting or attacking someone on purpose with or without using an object or weapon. In LSYPE1, it includes any of the following: vandalising public property; shoplifting; graffitiing on walls; fighting or public disturbance.
h. Measured at age 15/16 (wave 3) in LSYPE2 and LSYPE1.
i. Categories differed slightly from stated at LSYPE2, as follows: Never; Once a month or less; 2-3 times a month; 2 or more times a week.
j. Measured at age 16/17 (wave 4) in LSYPE2 and LSYPE1.
k. In LSYPE2, indicates whether young person has been a carer at age 16/17 (wave 4) only. In LSYPE1, indicates whether young person has been a carer at age 16/17 (wave 4) or age 17/18 (wave 5).

Notes:

1. Data are unweighted.
2. The n for the excluded sample refers to the maximum possible number of participants. Due to the nature of this sample having been excluded due to missing data, the n differs for each variable.

Acronyms: GCE: General Certificate of Education; GCSE: General Certificate of Secondary Education; A Level: Advanced Level.

References

1 Mansournia MA, Altman DG. Population attributable fraction. *BMJ* 2018; : k757.

2 Haneuse S, VanderWeele TJ, Arterburn D. Using the E-Value to Assess the Potential Effect of Unmeasured Confounding in Observational Studies. *JAMA* 2019; **321**: 602.
